# Health-related quality of life, direct medical and societal costs among children with moderate or severe haemophilia in Europe: multivariable models of the CHESS-PAEDs study

**DOI:** 10.1186/s13023-022-02301-0

**Published:** 2022-04-04

**Authors:** Idaira Rodriguez-Santana, Pronabesh DasMahapatra, Tom Burke, Zalmai Hakimi, José Bartelt-Hofer, Jameel Nazir, Jamie O’Hara

**Affiliations:** 1 HCD Economics, Daresbury, UK; 2 Sanofi Genzyme, Cambridge, MA USA; 3 University of Chester, Chester, UK; 4 Sobi, Stockholm, Sweden; 5 Sanofi, Chilly-Marzin, France

**Keywords:** Haemophilia A, Haemophilia B, Direct medical costs, Societal costs, Productivity, Quality of life, Children

## Abstract

**Background:**

Haemophilia bears substantial humanistic and economic burden on children and their caregivers. Characterising the differential impact of severe versus moderate paediatric haemophilia is important for clinical and health policy decisions. We analysed health-related quality of life (HRQoL), annual direct medical (excluding factor treatment costs), non-medical and societal costs among children and adolescents with moderate and severe haemophilia A or B without inhibitors from the European CHESS-PAEDs study. Information was reported by physicians and caregivers; patients aged ≥ 8 years self-reported their HRQoL. Descriptive statistics summarised demographic and clinical characteristics, costs, and HRQoL scores (EQ-5D-Y). Regression models estimated differences in HRQoL and costs for moderate versus severe haemophilia adjusting for age, body mass index z-score, country, number of comorbidities, and weight-adjusted annual clotting factor consumption.

**Results:**

The analytic sample comprised 794 patients with a mean age of 10.5 years; most had haemophilia A (79%) and 58% had severe haemophilia. Mean predicted direct medical costs in moderate patients were two-thirds of the predicted costs for severe disease (€3065 vs. €2047; *p* < 0.001; N = 794), while societal costs were more than half of the predicted costs for children with severe haemophilia (€6950 vs. €3666; *p* < 0.001; N = 220). Mean predicted HRQoL scores were 0.74 and 0.69 for moderate and severe disease, respectively (*p* < 0.05; N = 185).

**Conclusion:**

Children with haemophilia and their caregivers displayed a significant economic and humanistic burden. While severe patients showed the highest direct medical and societal costs, and worse HRQoL, the burden of moderate haemophilia on its own was substantial and far from negligible.

**Supplementary Information:**

The online version contains supplementary material available at 10.1186/s13023-022-02301-0.

## Background

Haemophilia is an inherited lifelong bleeding disorder characterised by inadequate clotting factor VIII in the case of haemophilia A or factor IX for haemophilia B, affecting primarily males. Haemophilia A has a substantially higher prevalence than haemophilia B—approximately 17.1 versus 3.8 cases per 100,000 males, respectively [1]. The severity of haemophilia is determined by the patient’s level of clotting factor activity, where those with < 1 IU/dL are considered to have severe disease, 1 to 5 IU/dL are considered moderate, and > 5 to < 40 IU/dL are considered mild [2]. Among people with haemophilia, 35.1% and 29.1% are diagnosed severe for Haemophilia A and B respectively [1]. Severe disease is associated with greater pain and disability from more frequent bleeding events, which can cause faster joint deterioration and associated sequelae [3, 4].

Paediatric haemophilia confers a burden not only on the children and adolescents, but also on their caregivers, including both psychosocial and financial challenges [5]. The care and treatment requirements for children with haemophilia are significant, including frequent intravenous (IV) infusions of factor replacement therapy (sometimes administered through central venous access devices with the consequent higher risk of infection and thrombosis [6]), frequent physician and hospital visits, and additional care and supervision needs. Parents of younger children with haemophilia have been shown to bear a greater burden than their peers particularly when additional clinical factors are present, such as inhibitors [7]. Therefore, characterising the impact of paediatric haemophilia on health-related quality of life (HRQoL) and societal costs should account for the perspectives of both the children and their caregivers.

Understanding the economic impact of paediatric haemophilia requires a broader view of the care to account for haemophilia-related direct medical costs from healthcare resource use, indirect costs such as for medical equipment and professional caregiving help, and societal costs such as diminished work productivity [8, 9]. We excluded the costs of factor replacement therapy from the direct medical cost outcome because treatment costs are known to account for the vast majority of total costs. In order to address a gap in the literature, this study focused specifically on estimating the impact of haemophilia severity on all other medical costs. Factor treatment have been largely documented in other studies and systematic reviews [9–11]. While research in this area is ongoing, there remains a need to better understand the differential impact of moderate and severe haemophilia in the paediatric population.

We conducted an analysis of the ‘Cost of Haemophilia across Europe: a Socioeconomic Survey in a Paediatric Population’ study (CHESS PAEDs) to quantify the differential direct medical and societal costs (including caregiver burden), and patient humanistic burden (HRQoL), of moderate in relation to severe disease paediatric patients with haemophilia A or B without inhibitors.

## Results

### Descriptive assessment of patient characteristics, costs, and HRQoL scores

The CHESS-PAEDs data set contained a total of 991 patients managed by 101 haematologists and haematology healthcare providers. Patients with current inhibitors (*n* = 146) and observations identified as outliers according to *Cook’s distance* [12] were excluded from the analysis (*n* = 43). Data pertaining to direct costs were available for 794 patients, of whom 28% (*n* = 220) had available information to calculate societal costs and 23% (*n* = 185) reported HRQoL outcomes (EQ-5D-Y). Demographic and clinical characteristics were generally similar across the total sample and sub-samples, with some exceptions related to clinical manifestations of haemophilia (Table 1). Overall, according to Direct Cost sample (*n* = 794), most patients (79%) had haemophilia A, most had severe haemophilia of either type (58%), and 76% were receiving a prophylaxis treatment regimen. The mean age of the total sample was 10.5 years (age range 1–17). Most patients had no comorbidities (82%), 1 to 5 bleeds per year (77%), and did not present any problem joints (PJ) (87%). Distribution of participants across the EU5 countries was relatively even.

**Table 1:** Patient demographic and clinical characteristics of each analytic sample

Characteristic	Direct costs Sample *n* = 794	Societal costs Sample *n* = 220	HRQoL (EQ-5D-Y) Sample *n* = 185
Age, mean (SD)	10.5 (4.74)	10.2 (4.58)	11.2 (3.79)
BMI z-score, mean (SD)	0.6 (1.59)	0.4 (1.46)	0.4 (1.39)
**BMI, *n* (%)**			
Underweight	24 (3)	12 (5)	8 (4)
Normal weight	474 (60)	132 (60)	113 (61)
Overweight	167 (21)	41 (19)	34 (18)
Obese	129 (16)	35 (16)	30 (16)
**Country, *n* (%)**			
Germany	131 (16)	55 (25)	49 (26)
Spain	182 (23)	40 (18)	27 (15)
France	171 (22)	40 (18)	30 (16)
Italy	197 (25)	38 (17)	35 (19)
United Kingdom	113 (14)	47 (21)	44 (24)
**Haemophilia type, *n* (%)**			
A	627 (79)	168 (76)	143 (75)
B	167 (21)	52 (24)	47 (25)
**Severity, *n* (%)**			
Moderate	331 (42)	58 (26)	43 (23)
Severe	463 (58)	162 (74)	142 (77)
**Comorbidities, *n* (%)**			
0	648 (82)	178 (81)	151 (82)
1	103 (13)	25 (11)	21 (11)
≥ 2	43 (5)	17 (8)	13 (7)
Treatment by severity*, *n* (%)			
*** Overall***	***n* = 794**	***n* = 220**	***n* = 185**
No treatment	92 (12)	12 (5)	8 (4)
On-demand	95 (12)	18 (8)	17 (9)
Prophylaxis	607 (76)	190 (86)	160 (86)
*** Moderate haemophilia***	***n* = 332**	***n* = 58**	***n* = 43**
No treatment	91 (27)	12 (21)	8 (19)
On-demand	48 (15)	1 (2)	3 (7)
Prophylaxis	192 (58)	45 (78)	32 (74)
*** Severe haemophilia***	***n* = 462**	***n* = 162**	***n* = 142**
No treatment	1 (0.2)	0	0
On-demand	47 (10)	17 (10)	14 (10)
Prophylaxis	415 (90)	145 (90)	128 (90)
**Annual bleeding rate, *n* (%)**			
** Overall**	***n* = 794**	***n* = 220**	***n* = 185**
0	119 (15)	14 (6)	12 (6)
1–5	609 (77)	182 (83)	152 (82)
> 5	66 (8)	24 (11)	21 (11)
** Moderate**	***n* = 331**	***n* = 58**	***n* = 43**
0	59 (18)	3 (5)	3 (7)
1–5	255 (77)	50 (86)	35 (81)
> 5	17 (5)	5 (9)	5 (12)
** Severe**	***n* = 463**	***n* = 162**	***n* = 142**
0	60 (13)	11 (7)	9 (6)
1–5	354 (76)	132 (81)	117 (82)
> 5	49 (11)	19 (12)	16 (11)
**Number of problem joints, *n* (%)**			
** Overall**	***n* = 794**	***n* = 220**	***n* = 185**
0	694 (87)	177 (80)	151 (82)
≥ 1	100 (13)	43 (20)	34 (18)
** Moderate**	***n* = 331**	***n* = 58**	***n* = 43**
0	299 (90)	48 (83)	37 (86)
≥ 1	32 (10)	10 (17)	6 (14)
** Severe**	***n* = 463**	***n* = 162**	***n* = 142**
0	395 (85)	129 (80)	114 (80)
≥ 1	68 (15)	33 (20)	30 (20)

Totals may not sum to 100% due to rounding

*BMI* body mass index, *SD* standard deviation

*“No treatment” category could include patients treated with alternative therapies such us desmopressin or antifibrinolytics

**Fig. 1:**
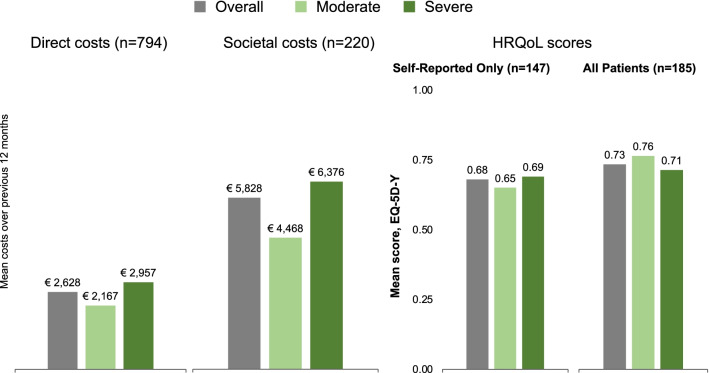
Summary of descriptive costs and HRQoL overall and by level of haemophilia severity

In the descriptive assessment, the mean annual direct medical costs excluding haemophilia treatment costs across all levels of severity were €2628 (standard deviation [SD], €3274), and unadjusted mean annual societal costs were €5,873 (SD, €8415). The mean HRQoL score was 0.73 (SD, 0.19). Participants with severe haemophilia appeared to have on average greater direct medical and societal costs, and worse HRQoL scores (all patients), than their peers with moderate disease as shown in Fig. 1.

The descriptive assessment of costs and HRQoL scores by treatment strategy showed the highest costs and lowest HRQoL scores for patients with severe haemophilia who were receiving an on-demand treatment regimen (Additional file 1: Appendix Table A1). Among participants receiving prophylaxis, those with severe disease had slightly higher unadjusted mean annual direct medical costs than those with moderate disease (€2821 and €2372, respectively) with slightly lower HRQoL scores (0.73 and 0.75, respectively). Substantial differences in the descriptive assessment of costs by country were apparent, where direct and societal costs were highest in Spain (€5310 and €13,592) and the United Kingdom (€5311 and €10,691). Direct and societal costs were expectedly higher, and HRQoL scores lower, among patients with higher annualised bleeding rates and more problem joints, where direct costs were 5 times higher with ≥ 5 bleeds/year versus zero bleeds/year and societal costs were 10 times higher (Additional file 1: Appendix Table A3). Mean HRQoL scores were approximately 10% lower with ≥ 5 versus zero bleeds/year (0.70 vs. 0.78, respectively; Additional file 1: Appendix Table A3).

#### Regression analysis of direct medical costs and societal costs

**Fig. 2:**
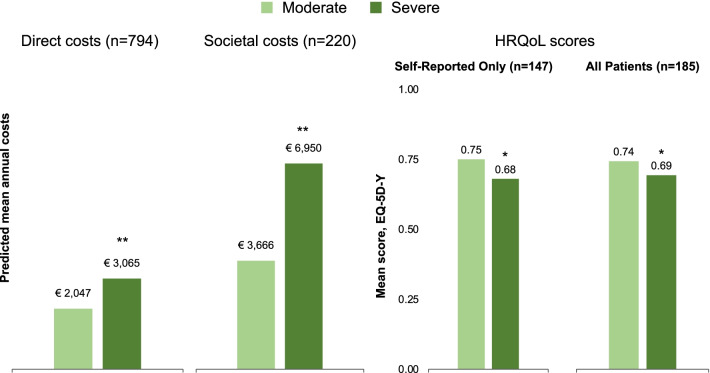
Predicted direct and societal costs and HRQoL (EQ-5D-Y) overall and by severity. *p < 0.05, **p < 0.001 vs. moderate disease. All models were adjusted for haemophilia severity, age, BMI z-scores, country, comorbidities, and total weight-adjusted factor consumption. Direct medical and societal costs were captured at the patient level for a period of 12 months

The regression analysis of both direct medical and societal costs showed significantly higher costs for severe versus moderate disease patients (both *p* < 0.001) when controlling for age, body mass index (BMI) z-scores, country, number of comorbidities, and total weight-adjusted annual factor consumption (Model 4). The predicted direct medical costs for moderate and severe disease were €2047 and €3065, respectively, and predicted societal costs were €3666 and €6950, respectively (Fig. 2). The average marginal effects (AME) estimates showed mean incremental annual direct medical costs of €1018 and mean incremental annual societal costs of €3284 with severe versus moderate disease (Table 2). Higher BMI z-score (distance from the mean BMI, *p* < 0.05) and having ≥ 2 comorbidities (*p* < 0.001) were significant predictors of higher direct medical costs. Having ≥ 2 comorbidities was also a significant predictor of higher societal costs (*p* < 0.05). Substantial between-country differences in both direct medical and societal costs were observed, as expected. Annual weight-adjusted clotting factor treatment consumption had a near-zero effect that was not statistically significant in either cost model. All model estimates and goodness of fit results are provided in Additional file 1: Appendix Table A2.

**Table 2:** Average marginal effects (AME) for annual direct medical costs, societal costs and HRQoL scores (EQ-5D-Y)

Parameter	Direct costs Sample *n* = 794	Societal costs Sample *n* = 220	HRQoL *Self-reported only* Sample (*n* = 147)	HRQoL *All patients* Sample (*n* = 185)
Severe vs. Moderate	€1018, *p* < 0.001	€3284, *p* < 0.001	−0.07, *p* < 0.05	−0.06, *p* < 0.05
Age, years	€ −5.71	€ −76.27	0.00	0.00
BMI z-score	€72.98, *p* < 0.05	€45.02	−0.03, *p* < 0.05	−0.01
**Country, vs. Germany**				
France	€194, *p* < 0.05	€384	0.06	0.06, *p* < 0.05
Italy	€229, *p* < 0.01	€ −139	−0.04	0.02
Spain	€4465, *p* < 0.001	€12,093, *p* < 0.001	0.08, *p* < 0.05	0.04
United Kingdom	€3868, *p* < 0.001	€8349, *p* < 0.001	0.10, *p* < 0.01	0.07, *p* < 0.05
**Comorbidities, vs. 0**				
1	€320	€940	−0.08	−0.01
≥ 2	€1393, *p* < 0.001	€2867	−0.182, *p* < 0.05	−0.18, *p* < 0.05
Annual factor consumption (IU/kg)	€0.01	€0.09	0.00	0.00
Caregiver proxy indicator	Not applicable	Not applicable	Not applicable	−0.021 (−0.36)

Statistical significance is indicated in italics: *p* < 0.001; *p* < 0.01; *p* < 0.05.* p* values are only provided for statistically significant AMEs

All models were adjusted for haemophilia severity (base outcome: moderate), age, BMI z-scores, country (base outcome: Germany), comorbidities (base outcome; zero comorbidities), and total weight-adjusted factor consumption

Direct medical and societal costs were captured at the patient level for a period of 12 months

#### Regression analyses of HRQoL

The regression analyses in the *Self-Reported Only* sample (patients aged 8–17 years, *n* = 147) and *All Patients* sample (that also includes caregiver proxy responses, *n* = 185) controlled for haemophilia severity, age, BMI z-scores, country, comorbidities, and total weight-adjusted factor consumption (Model 4). Results from the *Self-Reported Only* sample showed significantly worse HRQoL scores for patients with severe versus moderate disease (−0.07, *p* < 0.05; Table 2). The mean predicted HRQoL score for patients with moderate haemophilia was 0.75 (95% confidence interval [CI]: 0.69–0.80) and 0.68 (95% CI: 0.65–0.71; 9% lower) for patients with severe disease (Fig. 2). The AME estimates showed that patients with higher BMI-z scores (i.e., larger dispersion from the BMI sex–age group average) and ≥ 2 comorbidities contributed significant negative effects of −0.03 and −0.18 to the predicted HRQoL score, respectively (both *p* < 0.05). The estimate for annual weight-adjusted clotting factor treatment consumption was not statistically significant and very close to zero.

Overall findings were similar for the *All Patients* sample where those with severe haemophilia showed worse HRQoL scores (−0.06; *p* < 0.05) than those with moderate disease. The proxy indicator variable suggested that caregiver-reported EQ-5D-Y scores were lower than the children self-reported values (−0.02), but this was not statistically significant. Mean predicted HRQoL scores were 0.74 (95% CI: 0.69–0.79) and 0.69 (0.66–0.71) for moderate and severe disease, respectively (a 7% difference). The AME estimates showed a significant negative impact of having ≥ 2 comorbidities (−0.21, *p* < 0.05) on predicted HRQoL score. As with the *Self-Reported Only* sample, the estimate for annual adjusted factor consumption was approximately zero and not statistically significant. All model estimates and goodness of fit results are provided in Additional file 1: Appendix Table A2.

## Discussion

This analysis of the European CHESS-PAEDs study cohort quantified the economic and humanistic burden of haemophilia across levels of severity in children and adolescents without inhibitors. Disease severity was a significant predictor of increasing direct medical and societal costs and worse HRQoL scores.

While all outcomes were the least favourable for patients with severe disease, a significant humanistic and economic burden was also observed among those with moderate disease. Mean predicted direct costs in moderate patients were two-thirds of the predicted costs for patients with severe disease, while societal costs were more than half of the predicted costs for children with severe haemophilia. Although literature on children health spending is scarce, a report for the English paediatric population shows that it was estimated at £800 (2015–2016 data) [13], which is considerably below the direct medical costs observed in moderate and severe patients in CHESS-PAEDS.

Mean predicted EQ-5D-Y scores were 7% lower for severe versus moderate patients and 9% lower in the self-reported EQ-5D-Y sample for older children. In the absence of a published value set for the child/adolescent EQ-5D-Y, the published mean HRQoL population norms for 18- to 24-year-olds in the same European countries, ranging from 0.93 in the UK to 0.97 in Italy and Spain [14], are higher than those predicted in our ≤ 17-year-old cohort (0.71 and 0.76 for moderate and severe disease, respectively).

Clinical indicators such as greater BMI compared to peers and having ≥ 2 comorbidities (most common comorbidities were attention deficit, obesity and anxiety) also contributed to higher costs, as shown by regression estimates. We observed substantial differences in costs and HRQoL scores across the participating countries, likely attributable to differences in healthcare delivery and haemophilia management strategies, formal and informal caregiving practices, culture, and other societal factors. The descriptive assessments suggested lower direct medical and societal costs, and higher HRQoL scores, for patients with severe haemophilia receiving prophylaxis rather than an on-demand treatment regimen, which is consistent with published reports of long-term treatment outcomes for adult patients [15–17]. Such differences appeared negligible among patients with moderate disease. The effect of the proxy measure for treatment in the regression models (mean annual weight-adjusted clotting factor consumption, was negligible. This is probably due to opposing directional influences where more “complex” patients (more severe disease and more complications) tend to require more factor replacement therapy, yet greater factor consumption associated with prophylaxis can prevent complications and their related costs and consequences.

There were comparable mean annual bleeding rates and number of problem joints per year across moderate and severe patients (see Table 1), despite much larger proportions of severe than moderate patients being on a prophylaxis regimen (90% and 58%, respectively). These figures may suggest a substantial unmet treatment need for patients with moderate disease.

The findings of this study should be interpreted in the context of certain strengths and limitations. This was a retrospective analysis of an existing observational data set constructed from questionnaires completed by caregivers, patients and physicians. While patient-reported outcomes are particularly valuable in the context of burdensome, lifelong conditions such as haemophilia, data collection may have been influenced by a selection bias in participation and completion of the questionnaires. We reported descriptive results as reported among those participating in the CHESS-PAEDs study and performed rigorous regression analyses to adjust for relevant covariates. However, it is possible that unmeasured factors may have had an impact in the estimation results, which is a common limitation of empirical studies. The recording of clinical outcomes such as bleeding events and joint metrics from patients’ medical charts may be more straightforward than a formal clinical diagnosis, which requires imaging studies. Finally, our use of the child/adolescent version of the EQ-5D-Y was appropriate for this patient population, though there is no country-level normative value set available for the EQ-5D-Y to provide a closer contextual interpretation of our findings.

## Conclusions

Our findings have reinforced the humanistic and economic burden of moderate and severe haemophilia on paediatric patients without inhibitors and their caregivers in the European setting. While our work illustrates a significant differential burden of severe versus moderate haemophilia in terms of annual direct medical costs, societal costs, and patient HRQoL scores, the impact of moderate haemophilia on its own was substantial. Descriptive findings from this cohort also suggested a notable unmet treatment need (factor therapy) among patients with moderate haemophilia, whose frequency of annual bleeds was similar to that of patients with severe disease, possibly due in part to a high proportion of patients being untreated or receiving on demand therapy. This study may be useful for clinical and public health applications where the estimation of disease burden by severity levels may be lacking direct recent research and evidence. Future work may focus on children with moderate haemophilia to better understand the treatment patterns and disease burden experienced by these patients and their caregivers.

## Methods

We identified predictors of HRQoL and costs by severity of haemophilia using responses from participants in the CHESS-PAEDs study, which is a retrospective, cross-sectional study of male children and adolescents who fulfil the following inclusion criteria: older than 12 months old and up to 17 years of age; have moderate (clotting factor 1–5%) or severe (clotting factor< 1%) haemophilia A or B; and live in France, Germany, Italy, Spain, or the United Kingdom. CHESS-PAEDs data includes patients with or without inhibitors; however, this analysis was limited to those without inhibitors to factor therapy. Exclusion criteria were: mild disease (clotting factor > 5%), acquired haemophilia and other haemophilia subtypes. Physicians treating haemophilia invited the next 8 to 16 consulting paediatric patients who met the inclusion criteria to participate in the study. The physicians completed a web-based form that was based on the participant’s medical history and consultations from their medical records. The form included information on demographic and clinical characteristics as well as haemophilia-related direct medical costs. Caregivers completed questionnaires related to non-medical haemophilia-related direct costs, indirect costs and patient HRQoL. Physician reported record form was available for all participants, whilst the caregiver/patient questionnaire was returned on a voluntary basis. The CHESS-PAEDs data was collected between December 2017 and March 2018 and belongs to the CHESS family of datasets [18, 19].

### Outcome measures

We sought to understand the differential impact of the level of haemophilia severity (moderate or severe) on participants’ HRQoL, direct medical and societal costs. HRQoL was measured using the child/adolescent version of the EQ-5D-3L (EQ-5D-Y; www.euroqol.org), which contains five dimensions (mobility, selfcare, performance of usually activities, pain/discomfort, and anxiety/depression) and three levels (no problems, some problems, extreme problems). Participants aged 8 to 17 completed the EQ-5D-Y for themselves. For children up to 7 years old, caregivers completed the EQ-5D-Y Proxy 1 to provide responses from the caregiver’s perspective on the child’s health status. A health state index utility score was derived from an amalgam of responses across the five domains, with scores ranging from 0 (equivalent to “dead”) to 1 (“perfect health”), though scores of less than zero (“worse than dead”) were possible [20]. UK population norms were used across all samples for comparability purposes.

Haemophilia-related direct medical costs reported by physicians included costs related to acute events, physician consultation visits, hospitalisations, surgical procedures, tests and examinations, assistive medical devices (e.g., crutches), over-the-counter self-medication, and costs for professional caregiving (time and hourly cost). Direct non-medical costs included travel expenses for haemophilia-related care, qualifying government support, and alternative therapies. Work productivity impairment costs for caregivers was derived from hours worked per week, absenteeism, informal care costs, and early retirement. Societal costs were defined as the sum of all direct medical, non-medical, and work productivity costs. All costs were captured at the patient level and covered a 12-month period (costing details in Additional file 1: Appendix Table A3). The costs of factor replacement therapy and non-haemophilia-related costs were excluded.

### Statistical analysis

Descriptive statistics summarised demographic and clinical characteristics for the three analytic samples of patients with information on (1) direct medical costs; (2) societal costs; and (3) HRQoL scores. Outcomes were assessed overall and by relevant covariates, including haemophilia type (A or B), physician-reported haemophilia severity based on endogenous factor VIII or IX values for moderate (1–5%) or severe (< 1%), age, body mass index z-score (a measure of relative BMI adjusted for child age and sex relative to the Centers for Disease Control and Prevention [CDC] growth chart [21]), country, haemophilia treatment strategy (no treatment, on-demand, or prophylaxis), annualised clotting factor consumption (IU/kg), number of comorbidities (excluding haemophilia-related conditions; 0, 1, ≥ 2, complete list of comorbidities in Additional file 1: Appendix Table A2), annual bleeding rate (ABR; 0, 1–5, ≥ 5 bleeds in the previous 12 months), and number of problem joints (PJ; defined as chronic joint pain and/or limited range of movement due to compromised joint integrity, such as chronic synovitis and/or haemophilic arthropathy; 0 or ≥ 1 [22]). Haemophilia treatment strategy was based on the physician-reported total clotting factor usage (IU) in the previous 12 months. Alternative treatments such as desmopressin were not included. Details of the control variables are provided in Additional file 1: Appendix Table A4.

Regression models were developed to estimate cost outcomes for patients with moderate versus severe haemophilia using a generalised linear model (GLM) with Gamma distribution and log-link function. The AME were computed to ascertain the effect of each covariate on the outcome of interest. Four models were tested for each analytic sample, controlling for (1) severity only; (2) severity, age, BMI z-scores, and country; (3) model 2 plus number of comorbidities; and (4) model 3 plus total weight-adjusted factor consumption. Total factor consumption was used as a proxy for treatment strategy and to account for access to clotting factor treatment. Ultimately, the categorical value for type of haemophilia treatment (prophylaxis, on-demand, or no treatment) was discarded due to multicollinearity issues (> 80% of patients with severe haemophilia were receiving a prophylaxis regimen). Clinical outcomes such as bleeding events and problem joints were not included in the regression models since there is a known positive correlation between such outcomes and haemophilia severity level. Model 4 demonstrated the best goodness of fit (lowest Akaike and Bayesian information criteria) and was used for all analyses.

Differences in EQ-5D-Y scores across severity groups were estimated using a Tobit model bounded between −0.594 and 1.0; AME were used to show the effect of the covariate on the bounded outcome variable. The same four models described above were tested and model 4 demonstrated the best goodness of fit. We conducted the HRQoL analyses in two samples based on the completion mode of the EQ-5D-Y instrument. The “*Self-Reported Only*” sample included only children who provided all EQ-5D-Y responses for themselves (aged 8–17 years). The “*All Children*” sample comprised all children including those whose caregivers provided all or some of the responses as a proxy for the child.

Statistical significance was determined at the 5% alpha level (*p* < 0.05) for all analyses. No imputation of missing values was performed, and patients with missing responses were excluded from the analysis. All analyses were performed using STATA® 16 (StataCorp LLC, College Station, Texas; www.stata.com).

## Supplementary Information

**Additional file 1**. **Table A1**: Summary of costs and health status scores by demographic and clinical covariates. **Table A2**: Regression model results (parameter estimate with *t* statistic and *p* value). **Table A3**: Cost components used in the CHESS-PAEDs study. **Table A4**: Physician-reported control variable definitions

## Data Availability

The data that support the findings of this study may be available from HCD Economics, Ltd but restrictions apply to the availability of these data, which were used under license for the current study, and so are not publicly available. Data may be available from the authors upon reasonable request and with permission of HCD Economics Ltd.

## Abbreviations

ABR: Annual bleeding rate
AME: Average marginal effects
BMI: Body mass index
CDC: Centers for Disease Control and Prevention
CHESS-PAED: Cost of Haemophilia across Europe: a Socioeconomic Survey in a Paediatric Population
CI: Confidence interval
GLM: Generalised linear model
HRQoL: Health-related quality of life
IV: Intravenous
PJ: Problem joints
SD: Standard deviation
