# Supplementary material for: Health-related quality of life, direct medical and societal costs among children with moderate or severe haemophilia in Europe: multivariable models of the CHESS-PAEDs study
Source: Orphanet J Rare Dis. 2022 Apr 4;17:150. doi: 10.1186/s13023-022-02301-0 (PMC8981697; doi:10.1186/s13023-022-02301-0)
Supplement: Supplementary file 1 — Additional file 1. Table A1: Summary of costs and health status scores by demographic and clinical covariates. Table A2: Regression model results (parameter estimate with t statistic and p value). Table A3: Cost components used in the CHESS-PAEDs study. Table A4: Physician-reported control variable definitions [file 13023_2022_2301_MOESM1_ESM.docx]

APPENDIX

Table A1. Cost components used in the CHESS-PAEDs study

| **Outcome** | **Component category** | **Measured element** |
| --- | --- | --- |
| **Direct medical costs (excluding factor replacement therapy costs)** | Hospitalisations (Reported by the treating physician) | Day case |
|  |  | Outpatient (ie, for planned treatments) |
|  |  | Inpatient, including length of stay |
|  | Surgical procedures (Reported by the treating physician) | Number and type of surgeries |
|  |  | Length of stay |
|  |  | Time spent in intensive care |
|  | Consultant visits (Reported by the treating physician) | Haematologist |
|  |  | Other specialties |
|  | Tests and examinations (Reported by the treating physician) | Blood tests |
|  |  | Other tests and examinations |
|  | Professional caregiver (Reported by patient/caregiver) | Hourly wage |
|  |  | Hours per week |
| **Direct non-medical costs** | Alternative and Complimentary Therapies (Reported by patient/caregiver) | Number of visits |
|  |  | Cost per session |
|  | Travel costs (Reported by patient/caregiver) | Car |
|  |  | Public transport |
|  | Requirement for aids / equipment (Reported by patient/caregiver) | Walking aids |
|  |  | Home adjustments |
|  | Transfer Payments (Reported by patient) | Entitlement per month |
| **Indirect costs** | Caregiver burden (Reported by caregiver) | Hours per week |

Note: Country-specific unit cost sources were used to cost direct medical costs, information about cost data sources is available under request. Information on direct non-medical costs and indirect costs were reported by patients/caregivers in the patient survey (country-average unit costs were used if patient information was not available.) The most recent estimate for each specific unit cost was used, all costs were converted to 2018 Euros.

Table A2. Physician-reported control variable definitions

| **Variable** | **Detail** |
| --- | --- |
| Haemophilia type | haemophilia A, haemophilia B |
| Severity | Severe (levels of factor VIII or IX <1%)  Moderate (levels between 1%-5%) |
| Bleed frequency | Annual bleed rate (ABR) derived from physician-reported major plus minor bleeds in the previous 12 months:  No Bleeds, 1-5 Bleeds, 5+ Bleeds. |
| Problem joints*  (areas of chronic synovitis) | Having chronic joint pain and/or limited range of movement due to compromised joint integrity (i.e. chronic synovitis and/or haemophilic arthropathy): None, ≥ 1  *Problem joint is a broader definition of joint morbidity, with respect to traditional measures solely focused on bleeding, e.g, target joint, with the objective to better characterise the burden of patients. |
| Therapy regimen | Prophylaxis, on-demand, or no treatment based on physician-reported total IU usage in the past 12 months.  No treatment category could include patients treated with alternative therapies such us desmopressin or antifibrinolytics. |
| Clotting factor consumption | Total IU/kg usage in the past 12 months (weight adjusted) |
| Country | France, Italy, Germany, Spain, United Kingdom |
| Age | Age 1-17 |
| Body mass index (kg/m^2^) z-score | Modified z-score BMI value (a measure of relative BMI adjusted for child age and sex relative to CDC growth chart) |
| Concomitant conditions | Number of comorbidities the patient is experiencing at index date (haemophilia-related comorbidities are excluded): None, One, Two or more  List of comorbid conditions: Attention Deficit (Hyperactivity) Disorder, Autism spectrum disorder, diabetes mellitus, obesity, anaemia, osteoarthritis, osteoporosis, Von Willebrand’s disease, gingivitis, other non-haemophilia-related disorder (open response field) |

Table A3. Summary of costs and health status scores by demographic and clinical covariates

| **Characteristic** | **Direct costs (€)**  **n=794** | **Societal costs (€)**  **n=220** | **HRQoL score**  **n=185** |
| --- | --- | --- | --- |
| **Country, mean (SD)**  Germany  Spain  France  Italy  United Kingdom | 900 (710)  5,310 (4,200)  1,171 (884)  1,024 (803)  5,311 (3,782) | 1,612 (2,284)  13,592 (10,608)  2,361 (1,941)  1,651 (2,369)  10,691 (10,255) | 0.70 (0.16)  0.70 (0.23)  0.77 (0.10)  0.72 (0.19)  0.75 (0.23) |
| **Comorbidities, n (%)**  0  1  ≥2 | 2,229 (2,781)  3,122 (2,749)  7,460 (4,709) | 4,595 (7,016)  8,886 (10,097)  14,817 (12,421) | 0.74 (0.17)  0.72 (0.17)  0.56 (0.32) |
| **Treatment, n (%)**  ***Overall***  No treatment  On-demand  Prophylaxis  ***Moderate***  No treatment  On-demand  Prophylaxis  ***Severe***  No treatment  On-demand  Prophylaxis | 1,836 (2,623)  3,157 (3,434)  2,665 (3,321)  1,836 (2,623)  2,128 (1,979)  2,371 (3,045)  –  3,518 (3,940)  2,821 (3,494) | 8,441 (8,842)  10,296 (12,507)  5,291 (7,798)  8,441 (8,842)  3,974 (4,089)  3,350 (4,225)  –  8,066 (10,736)  5,840 (8,562) | 0.73 (0.22)  0.75 (0.17)  0.72 (0.19)  0.73 (0.22)  0.87 (0.13)  0.75 (0.18)  –  0.68 (0.14)  0.73 (0.20) |
| **Annual bleeding rate, n (%)**  0  1 to 5  ≥5 | 1,219 (1,460)  2,611 (3,177)  5,327 (4,634) | 4,107 (7,629)  4,787 (6,316)  15,138 (15,054) | 0.78 (0.14)  0.72 (0.18)  0.70 (0.25) |
| **Number of problem joints, n (%)**  0  ≥1 | 2,253 (2,746)  5,229 (5,026) | 4,595 (6,828)  11,132 (11,774) | 0.74 (0.17)  0.64 (0.24) |

SD, standard deviation.

Direct medical and societal costs are captured at patient level for a period of 12 months.

Cost in Euros, year 2018.

Table A4. Regression model results (parameter estimate with *t* statistic and *P* value)

| **Parameter** | **Direct costs Sample**  **n=794** | **Societal costs Sample**  **n=220** | **HRQoL**  ***Self-reported only* Sample**  **(n=147)** | **HRQoL**  ***All patients* Sample**  **(n=185)** |
| --- | --- | --- | --- | --- |
| Severe vs Moderate | 0.404 (7.10), P<0.001 | 0.640 (4.14), P<0.001 | –0.099 (–1.99) , P<0.05 | –0.081 (–1.96) |
| Age, years | –0.002 (–0.41) | –0.013 (–0.89) | 0.001 (0.21) | 0.001 (0.24) |
| BMI z-score | 0.028 (2.47), P<0.05 | 0.008 (0.18) | –0.036 (–2.21) , P<0.05 | –0.014 (–1.29) |
| Country, vs Germany  France  Italy  Spain  United Kingdom | 0.194 (2.32), P<0.05  0.225 (2.65), P<0.05  1.777 (20.92), P<0.001  1.659 (17.24), P<0.001 | 0.195 (0.87)  –0.081 (–0.32)  2.052 (8.18) , P<0.001  1.738 (7.37) , P<0.001 | 0.079 (1.75)  –0.045 (–0.78)  0.113 (1.90)  0.143 (2.85) , P<0.01 | 0.077 (1.97)  0.024 (0.55)  0.050 (0.96)  0.100 (2.01) , P<0.05 |
| Caregiver proxy indicator | Not applicable | Not applicable | Not applicable | –0.021 (–0.36) |
| Comorbidities, vs 0  1  ≥2 | 0.125 (1.66)  0.456 (4.77), P<0.001 | 0.162 (0.68)  0.430 (2.01) , P<0.05 | –0.110 (–1.77)  –0.227 (–2.37) , P<0.05 | –0.011 (–0.23)  –0.212 (–0.23) , P<0.05 |
| Annual factor consumption (IU/kg) | 0.00000447 (0.70) | 0.0000149 (0.70) | –0.00000529 (–0.79) | 0.00000138 (0.23) |
| Constant  Observations  R-squared  RMSE | 6.505 (74.63), P<0.001  794  0.517  2295.91 | 7.015 (32.08) , P<0.001  220  0.438  6457.55 | 0.793 (8.33) , P<0.001  147  0.909  0.041, P<0.001 | 0.762 (8.77), P<0.001  185  1.061  0.0398, P<0.001 |

All models were adjusted for haemophilia severity (base outcome: moderate), age, BMI z-scores, country (base outcome: Germany), comorbidities (base outcome; zero comorbidities), and total weight-adjusted factor consumption.

Direct medical and societal costs were captured at the patient level for a period of 12 months.

Statistical significance is indicated in italics: *P<0.001; P<0.01; P<0.05*. *P>0.05* for all other differences.

¡
